# Characterization of sulfur-compound metabolism underlying wax-ester fermentation in *Euglena gracilis*

**DOI:** 10.1038/s41598-018-36600-z

**Published:** 2019-01-29

**Authors:** Koji Yamada, Tomoaki Nitta, Kohei Atsuji, Maeka Shiroyama, Komaki Inoue, Chieko Higuchi, Nobuko Nitta, Satoshi Oshiro, Keiichi Mochida, Osamu Iwata, Iwao Ohtsu, Kengo Suzuki

**Affiliations:** 1 euglena Co., Ltd., Tokyo, 108-0014 Japan; 2 Microalgae Production Control Technology Laboratory, RIKEN, Kanagawa, 230-0045 Japan; 3 Innovation Medical Research Institute, University of Tsukuba, Ibaraki, 305-8577 Japan; 4 Center for Sustainable Resource Science, RIKEN, Kanagawa, 230-0045 Japan; 5 Department of Bioresources Engineering, National Institute of Technology, Okinawa College, Okinawa, 905-2192 Japan; 6 Kihara Institute for Biological Research, Yokohama City University, Kanagawa, 244-0813 Japan; 7 Institute of Plant Science and Resources, Okayama University, Okayama, 710-0046 Japan

## Abstract

*Euglena gracilis* is a microalga, which has been used as a model organism for decades. Recent technological advances have enabled mass cultivation of this species for industrial applications such as feedstock in nutritional foods and cosmetics. *E. gracilis* degrades its storage polysaccharide (paramylon) under hypoxic conditions for energy acquisition by an oxygen-independent process and accumulates high amount of wax-ester as a by-product. Using this sequence of reactions referred to as wax-ester fermentation, *E. gracilis* is studied for its application in biofuel production. Although the wax-ester production pathway is well characterized, little is known regarding the biochemical reactions underlying the main metabolic route, especially, the existence of an unknown sulfur-compound metabolism implied by the nasty odor generation accompanying the wax-ester fermentation. In this study, we show sulfur-metabolomics of *E. gracilis* in aerobic and hypoxic conditions, to reveal the biochemical reactions that occur during wax-ester synthesis. Our results helped us in identifying hydrogen sulfide (H_2_S) as the nasty odor-producing component in wax-ester fermentation. In addition, the results indicate that glutathione and protein degrades during hypoxia, whereas cysteine, methionine, and their metabolites increase in the cells. This indicates that this shift of abundance in sulfur compounds is the cause of H_2_S synthesis.

## Introduction

Biofuel produced from algae is categorized as a third generation biofuel and is expected to attain high yields in the future^1^. The high potential of third generation biofuel is due to the diversity of algae in environment, the need of non-arable land for its growth, higher algal biomass and oil yields, which allows the biofuel production for various purpose^1^. Many algal species are already evaluated for its biomass productivity and studied for industrial application^2^. One such microalgae is *Euglena gracilis*, which is a species that is extensively used for decades as a model organism to study the mechanisms of photosynthesis and metabolism. *E. gracilis* is a unicellular and eukaryotic flagellate with secondary plastids for photosynthesis^3^. In hypoxia, *E. gracilis* produces wax-ester which includes myristyl myristate (C14:0-C14:0Alc) as a main component; this is suitable as a feedstock for bio-jet fuel^4,5^. The fast proliferation speed, tolerance to low pH (~3.5), and ease of inducing lipid production benefit the application of this species to biofuel production^6^. *E. gracilis* has already been industrially cultivated to be used as feedstock for functional foods and cosmetics^7^, and is further studied to be applied for biofuel production^8–10^.

*E. gracilis* produces wax-ester in hypoxia as a result of anaerobic synthesis of ATP by decomposition of paramylon^4,11^, a storage polysaccharide specific to *Euglena*^12^. In hypoxia, paramylon is degraded to glucose units and metabolized to pyruvate, which is oxidized by the O_2_-sensitive enzyme pyruvate:NADP^+^ oxidoreductase to produce acetyl-CoA in mitochondria^13^. The acetyl-CoA produced in mitochondria is used as C2 donor in a reversal reaction of beta-oxidation to form acyl-CoA^14^. Acyl-CoA is exported to the endoplasmic reticulum, reduced to fatty alcohol, and is esterified with another acyl-CoA to form wax-ester^15^. This series of processes is well characterized as wax-ester fermentation; however, other metabolic processes are surmised to progress in the background. Consistent with this, a recent study revealed that organic acids such as succinate and lactate are excreted outside the cells in hypoxia^16^. Although no report exists so far, wax-ester fermentation is empirically known to accompany the generation of a nasty odor, which is predicted to be of sulfur compounds, indicating that sulfur metabolism occurs at the background of wax-ester fermentation.

The sulfur metabolism includes a wide variety of reactions that form a complicated metabolic network^17^. L-cysteine plays a pivotal role in this network as cysteine is a metabolic hub, and its reduced sulfur and thiol groups are strongly nucleophilic. Animals require sulfur-containing amino acids as essential nutrients, while plants can assimilate inorganic sulfate (SO_4_^2−^) to form sulfide (S^2−^), which is incorporated in cysteine. In *E. gracilis*, although sulfate is assimilated, the reduction of sulfate does not occur in the chloroplasts; instead, it takes place in the mitochondria^18,19^. An important role of sulfur in biological systems is that it controls redox state in the cells; for example, glutathione (GSH), which is a tripeptide composed of glutamate, cysteine, and glycine, reduces the reactive oxygen species to protect cells from free radical damages. In this reaction, GSH is altered to an oxidized form of glutathione (GSSG), which is formed by a covalent disulfide bond between two GSHs and can be restored to separate GSHs by the activity of NADPH-glutathione reductase^17,20^. In healthy cells, more than 90% of the total glutathione is in the form of GSH, while most of the remaining glutathione is in the form of GSSG to balance the redox state in the cells^17,20^.

The recent development of a method for LC/MS has enabled analysis of sulfur metabolomics even in small sample volumes^21^. This method detects and compares around 87 chemical compounds related to sulfur metabolism so far. To achieve deeper understanding of wax-ester fermentation mechanism in *E. gracilis*, we studied the basis for nasty odor production during wax-ester fermentation by performing sulfur metabolomics. Our results show that the primal cause of nasty odor in wax-ester fermentation is the excretion of hydrogen sulfide (H_2_S). The synthesis of H_2_S in hypoxic conditions was not affected by the extracellular sulfate, indicating that it was not due to the sulfate-reducing respiration observed in sulfate-reducing bacteria^22^. In addition, the results indicate that the metabolism of sulfur-containing compounds is altered in hypoxic condition, suggesting a process of H_2_S synthesis from intracellular sulfur-containing compounds.

## Results

### Sulfur metabolomics of *E. gracilis* in aerobic condition and hypoxia

To study the regulation of sulfur-containing compounds in hypoxia, we conducted sulfur metabolomics of *E. gracilis* cells, and its extracellular medium in aerobic as well as in hypoxic conditions. The experiment was designed to analyze the samples which are hypoxically incubated for 24 hours as enough wax-ester accumulates in the first 24 hours^11^ and the cells start dying at that period (Supplementary Fig. 1). Specifically, after 3 days of heterotrophic cultivation of *E. gracilis* in triplicate, the samples were prepared by incubating the cells for 24 hours at four different conditions, viz. aerobic and hypoxic incubation in phosphate buffer, hypoxic incubation in phosphate buffer with the addition of 1 mM sulfate (SO_4_^2−^), and with the addition of 1 mM thiosulfate (S_2_O_3_^2−^) (Supplementary Fig. 2A,B). The cell pellet and supernatant were subjected to sulfur-metabolomics independently. In addition, the residual cells were dried and subjected to the quantification of lipid content to verify the progress of wax-ester production (Supplementary Fig. 2C,D). Among the sulfur compounds that can be analyzed with this method, 36 compounds were detected either in the cell pellet or supernatant (Supplementary Fig. 3).

### Generation of hydrogen sulfide from *E. gracilis* in hypoxia

**Figure 1:**
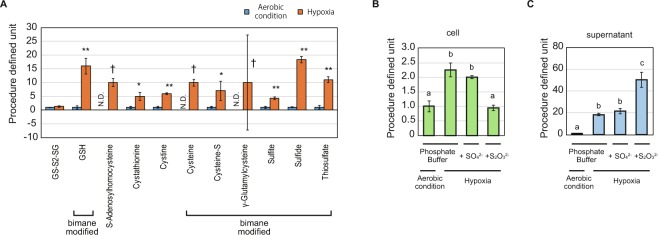
Upregulation of sulfide in hypoxia. (**A**) Upregulated extracellular sulfur-containing compounds in hypoxia. Eleven sulfur-containing compounds were identified as compounds upregulated in hypoxia. The data for both aerobic condition and hypoxia are derived from the cell supernatant in phosphate buffer medium. “Bimane modified” indicates the compounds detected as bimane-modified compounds. Procedure defined unit is calculated by normalizing the LC/MS signals to that of aerobic conditions for each compound. Error bars indicate SD. N.D. indicates that the compound was not detected in the condition. n = 3, *p < 0.05 t-test, **p < 0.05 t-test with Bonferroni’s correction, ^†^compounds were not detected in aerobic condition. (**B,C**). Detailed quantification of sulfide in cell pellet (**B**) and supernatant (**C**) for each condition is shown at the bottom of the graphs. Procedure defined unit is calculated by normalizing the LC/MS signals by that of the aerobic condition for each graph. Error bars indicate SD. n = 3, a, b, c: means with the different character indicates significant difference, p < 0.05 Tukey’s multiple test.

The cause of the nasty odor of *E. gracilis* in hypoxia was identified as hydrogen sulfide (H_2_S), by examining the result of sulfur metabolomics. Eighteen compounds were detected in the supernatant of any one of the aforementioned conditions (Supplementary Fig. 3). Among these compounds, 11 sulfur-containing compounds were increased by hypoxic conditioning in phosphate buffer (Fig. 1A). Sulfide, which includes H_2_S, hydrosulfide ion (HS^−^), and sulfide ion (S^2−^), was detected as the sole compound, which increased under hypoxia and could emit an odor. This indicates that H_2_S generation is the primary cause of the nasty odor of hypoxic-conditioned *E. gracilis* culture.

Our sulfur metabolomics data suggest that H_2_S synthesis is not due to sulfate-reducing respiration. Sulfate-reducing microorganisms respire in anaerobic conditions using sulfate instead of oxygen and generate H_2_S as the result of respiration^22,23^. To examine whether *E. gracilis* performs anaerobic respiration, we analyzed the sulfur metabolome data, to study the effect of sulfate addition to the hypoxic incubation (Fig. 1B,C). The addition of sulfate showed no effect on the value of sulfide, both in the cells and in the supernatant, thus suggesting that the generation of H_2_S is not a consequence of sulfate-reducing respiration. Instead, the results imply that sulfur-containing compounds in the cells are metabolized to synthesize H_2_S in hypoxia.

Our data also indicate that the generation of H_2_S was not sufficient for enhancing wax-ester production in hypoxia. We further examined the effect of thiosulfate, which is assimilated by microorganisms by a different pathway other than sulfate, and is easily metabolized to H_2_S in sulfur-redundant condition^24^ under hypoxia (Fig. 1B,C). The amount of sulfide in the cells unexpectedly decreased while that in the supernatant increased, suggesting that thiosulfate was aggressively metabolized to H_2_S and the synthesis of sulfide from intracellular sulfur-containing compounds was suppressed under hypoxia. The lipid productivity in hypoxic condition was affected by neither sulfate nor thiosulfate addition (Supplementary Fig. 2D). These results are consistent with the fact that *E. gracilis* does not use sulfate for anaerobic respiration. In addition, the results suggest that the generation of H_2_S is not directly linked to the wax-ester production process.

### Decrease of glutathione in hypoxic condition

**Figure 2:**
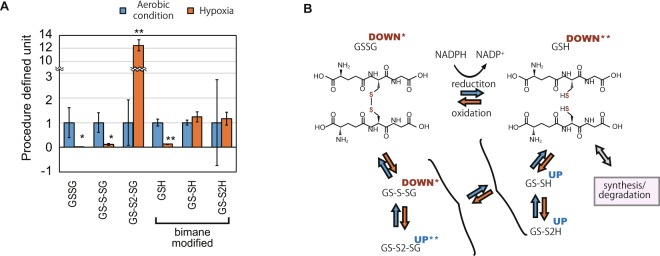
Decrease of total glutathione in hypoxia. (**A**) Relative quantification of glutathione (GSH)-related compounds in the cells in aerobic condition and hypoxia. The detected GSH compounds are reduced GSH, its oxidized form synthesized by covalent disulfide bond between two GSH molecules (GSSG), and their persulfidated derivatives GS-SH, GS-S2H, GS-S-SG, and GS-S2-SG. Procedure defined unit is calculated by normalizing the LC/MS signals to that of aerobic conditions for each compound. Error bars indicate SD. n = 3, *p < 0.05 t-test, **p < 0.05 t-test with Bonferroni’s correction. (**B**) An illustration of the reduction and oxidization network of glutathione. Red and blue arrows indicate the alteration in redox state of the compounds. The superscript for each compound indicates its upregulation (UP) or downregulation (DOWN) with or without a mark indicating the significant difference (*p < 0.05 t-test, **p < 0.05 t-test with Bonferroni’s correction).

Our quantification of GSH-related compounds indicates that they are degraded in hypoxic conditions with the shift to oxidized forms. To evaluate the redox status of the cells in hypoxic condition, we focused on the increase and decrease of GSH compounds in the sulfur metabolomics data (Fig. 2A,B). GSH and its major oxidized form GSSG decreased in hypoxic conditions, indicating that the total quantity of GSH-related compounds decreases. Although GS-S-SG, a persulfide form of GSSG, also decreased, another persulfide form of GSSG, i.e., GS-S2-SG, increased more than ten times. In addition, persulfide forms of GSH, GS-SH, and GS-S2H, did not decrease even when there was a decrease in total GSH compounds, thus suggesting that the ratio of persulfide forms among GSH compounds increased and the intracellular environment is in oxidized state. As GSH compounds are the most abundant low molecular weight thiol compounds synthesized in the cells, degradation of the compounds in hypoxic condition is a potential cause of H_2_S generation.

### Alteration of sulfur metabolism in hypoxia

**Figure 3:**
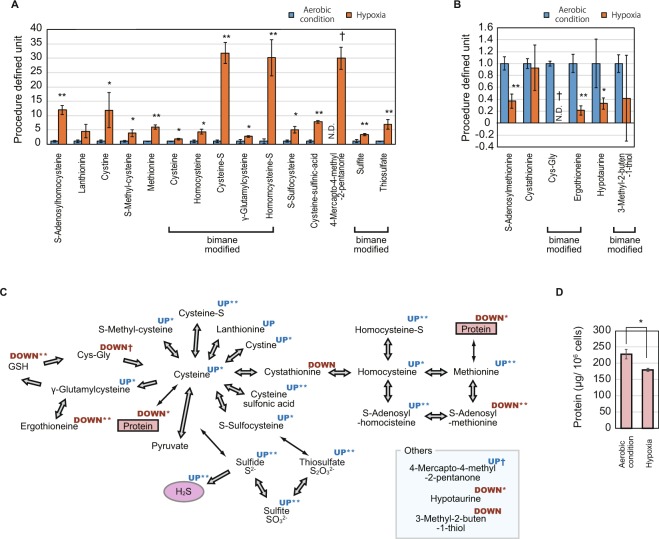
Increased and decreased sulfur compounds in hypoxia. (**A,B**) Relative quantification of increased (**A**) and decreased (**B**) sulfur-containing compounds in the cells in aerobic conditions and hypoxia. Procedure defined unit is calculated by normalizing the LC/MS signals to that of aerobic condition for each compound. Error bars indicate SD. N.D. indicates that the compound is not detected in the condition. n = 3, *p < 0.05 t-test, **p < 0.05 t-test with Bonferroni’s correction, ^†^compounds are not detected in either of the conditions. (**C**) An illustration of the metabolic network of sulfur compounds. The arrows between compounds indicate metabolic reactions. The superscript for each compound indicates the upregulation (UP) or downregulation (DOWN) of the compound with or without a mark indicating the significant difference (*p < 0.05 t-test, **p < 0.05 t-test with Bonferroni’s correction, ^†^compounds are not detected in either of condition). (**D**) Quantification of protein in the cells in aerobic conditions and hypoxia. Error bars indicate SD. n = 3, *p < 0.05 t-test.

Our sulfur metabolomics data indicate that cysteine and its metabolites increase in hypoxia, suggesting that H_2_S is generated through this alteration in metabolic activity. Among the 21 sulfur-containing compounds in the cells other than GSH and its derivatives, the quantity of the 15 and 6 compounds increased and decreased in hypoxia, respectively (Fig. 3A,B). Most of the metabolites of cysteine and methionine increased, suggesting that this upregulation was the primal source of H_2_S generation (Fig. 3C). The degradation of GSH compounds (Fig. 2A) contributes to this supply of cysteine, while another large source of sulfur-containing amino acids is protein in the cells. Our quantification of the protein in the cells revealed its decrease in hypoxia (Fig. 3D), suggesting that the protein acts as another supply source of sulfur-containing amino acids, which are, in turn, decomposed to generate H_2_S (Fig. 3C).

The analyses of the publicly available RNA-seq data of *E. gracilis* suggest that the amount of GSH is altered via the upregulation of GSH degrading genes. GSH is synthesized by a sequential reaction catalyzed by two enzymes, γ-glutamylcysteine synthetase (GCS) and GSH synthetase (GS), while its degradation is initiated by γ-glutamyltransferase (γ-GT) which removes a γ-Glutamyl residue from GSH^25^. Although the degradation of GSH by γ-GT primarily occur at the extracellular space in animal, recent studies showed that yeast possesses a γ-GT independent pathway to degrade GSH in the cell and uses the synthesized amino acid as nutrient. In the alternative pathway, GSH degradosome composed of Dug1p, Dug2p, and Dug3p participates^26^. By BLAST search of the previously reported transcriptome assembly data of *E gracilis*, GDJR00000000.1^27^, we found that the contigs, GDJR01097307.1, GDJR01026204.1, GDJR01056462.1, and GDJR01091960.1, encode the orthologues of GCS, GS, γ-GT, and GSH degrardosome proteins, respectively (Supplementary Fig. 4A). We further used the deposited RNA-seq data^27^ to estimate the expression abundance of each contig under aerobic and hypoxic conditions based on reads per million (RPM) value. The results indicate that the contigs of GCS and GSH degradosome are increased under hypoxic condition (Supplementary Fig. 4B). The upregulation of GCS is consistent with the increase of the intermediate, γ-glutamylcysteine, while the upregulation of GSH degradosome suggests that the GSH degradation in hypoxic condition is accomplished by this degradation pathway.

## Discussion

### Negative effect of H_2_S generation

The generation of H_2_S possibly hampers the progress of wax-ester fermentation in *E. gracilis* owing to its toxic effects. The generation of nasty odor in the course of wax-ester fermentation was empirically known; however, to the best of our knowledge, no details had been reported on this phenomenon so far. In this study, we have revealed the identity of nasty odor as H_2_S, which is generated through the degradation of GSH and protein. In the course of hypoxic conditioning for wax-ester production, the cells gradually start dying after 24 hours of exposure, which is a result of the shortage of polysaccharide as energy source. As H_2_S is known to damage cells by inhibiting cytochrome C oxidase and exposing the cells to oxidative stress^28^, our results suggest that the death of cells in wax-ester fermentation may be also partly due to the toxic effect of H_2_S. In that case, the productivity of wax ester may be improved by developing a method to suppress the H_2_S generation or removing it from the medium effectively.

In addition to the negative effects discussed above, H_2_S produces negative effects on the environment and on the value of defatted residue produced in a process for biofuel production. H_2_S is an undesirable byproduct as it possibly causes odor pollution and health problems. In addition, the reduction of GSH and protein along with the production of H_2_S detracts from the value of the defatted residue, which are to be used as feed and fertilizer^7^. The addition of thiosulfate to the hypoxic incubation enhanced the production of H_2_S outside the cells (Fig. 1C), while the lipid content was not affected by it (Supplemental Fig. 1C), suggesting that the conversion of thiosulfate to H_2_S is not sufficient to forward the wax-ester production process. In addition, although the degradation of glutathione and protein results in H_2_S synthesis in the background of wax-ester production (Figs 2A,B, 3C,D), it is not obvious whether this process is necessary for wax-ester production. Therefore, the suppression of H_2_S generation is an important criterion for future engineering to succeed in biofuel business using *E. gracilis*.

### Physiologic reason for the degradation of GSH and protein

The reduction of GSH-related compounds in hypoxia resembles the results reported in culture cells, suggesting that the reduction is a consequence of a widely conserved mechanism. In culture cells, GSH-related compounds are reduced in hypoxic conditions because of the reaction with reactive oxygen species synthesized in the mitochondrial electron transport chain and the prevention of new synthesis^29^. In addition to the reduction of GSH, our sulfur-metabolomics data show an increase in γ-glutamylcysteine, which is the precursor of GSH^17^, in *E. gracilis* cells under hypoxic conditions (Figs 2A,B, 3A,C). This result was consistent with our analysis of publicly available RNA-seq data which suggested that the enzyme for γ-glutamylcysteine production, GCS, is upregulated in hypoxia. In addition, GS was not downregulated in *E. gracilis* cells under hypoxic condition, suggesting that the mechanism to reduce GSH-related compounds in hypoxic condition is different between culture cells and *E. gracilis* cells. Our analysis also suggested that GSH degradosome are upregulated in hypoxic condition, suggesting that the decrease of GSH in hypoxic condition is due the action of this degradation pathway. Although GSH is present in cytosol, chloroplasts, and mitochondria, our result does not distinguish between the locations of the metabolites. In order to understand the metabolism of GSH in depth, quantification of GSH by compartmenting the organelle is required.

A conceivable reason for the degradation of GSH and protein is that the produced amino acids are required for energy acquisition by oxygen-independent manner. In the experiments, although the samples were shielded from light to prevent the synthesis of oxygen by photosynthesis and ensure hypoxia, the control group in aerobic condition was also shielded from light, indicating that the degradation of protein is not a result of dedifferentiation of chloroplast in the dark^30^. Instead, GSH and protein may be degraded for anaerobic catabolism of amino acids as well as polysaccharides for energy acquisition, such as that in anaerobic bacteria^31,32^. For cysteine, multiple degradation pathways which accompany the production of H_2_S are reported in a wide variety of organisms^33,34^, supporting the idea that the production of H_2_S in hypoxia-conditioned *E. gracilis* is a result of cysteine catabolism. As per this point of view, the introduction of transporters of cysteine or its metabolite to the cells will contribute to the reduction of H_2_S generation.

## Materials and Methods

### Strains and sample preparation

The *E. gracilis* (WT Z) strain provided by IAM (JAPAN) was used in this study. The *E. gracilis* Z strain was maintained in CM medium (pH 3.5)^35^, whereas the culture for sample production was performed in KH medium (pH 3.5)^36^. Pre-culture was conducted for a week in a conical flask, at a 200-mL scale, with rotary shaking at 100 rpm, constant temperature of 29 °C, and with constant illumination of 100 µmol/m^2^s. Then, the pre-culture was washed twice with KH medium and diluted to 4.5 × 10^6^ cells/mL to be used for the main culture. The main culture was conducted in triplicate at a 200-mL scale with the same conditions as the pre-culture. After 3 days of culture, each culture was washed twice with a phosphate buffer and divided evenly into four parts. Three of the four parts were suspended in 50 mL of phosphate buffer, phosphate buffer with 1 mM MgSO_4_, and phosphate buffer with 1 mM Na_2_S_2_O_3_ in 50 mL centrifuge tubes, respectively. These were subjected to hypoxic conditioning by screwing the cap tightly and by light shielding. The fourth part was suspended in 50 mL of phosphate buffer in a conical flask and was incubated at 29 °C with rotary shaking at 100 rpm and with light shielding. After 24 hours of incubation, samples at the four conditions, each in triplicate, were used for sulfur metabolomics and other analyses. Phosphate buffer (67 mmol/L, pH 6.8) was prepared, by using aqueous solutions of Na_2_HPO_4_ and KH_2_PO_4_.

### Sulfur metabolomics

Sulfur metabolomics was performed by Sulfur Index service in JAPAN (http://www.euglena.jp/sulfurindex/). Briefly, the sulfur-containing compounds in the samples were extracted by adding MeOH and were converted to fluorescent derivatives with mono-bromobimane. The samples were then dried, re-dissolved in water, and analyzed with sensitive LC-MS/MS as reported^21^. The MS signal was identified and quantified manually and was corrected with respect to the number of cells subjected to the sample preparation. Then, the value was normalized as shown in the legend of each graph.

### Quantification of lipid and protein content

The cell density and liquid volume of each leftover culture were preliminarily examined. Then, the cells were collected by centrifugation (1,000 *g*, 3 min) and dried in a freeze dryer (FDV-1200, EYELA). Approximately 10 mg and 50 mg samples of the dried cells were used for quantifying their intracellular protein and lipid content, respectively. Protein was extracted from the cells by suspending the dried cells in 200 μL of RIPA buffer (WAKO) and homogenizing it for 10 sec in a sonicator (UD-201, TOMY). After removing the debris by centrifugation (10,000 *g*, 5 min), the protein content was evaluated by using a BCA protein assay kit (Takara) following the manufacturer’s instructions. The neutral lipid was extracted from the dried cells by using n-hexane as a solvent^37^. The dried cells were suspended in 10 mL of n-hexane and were homogenized for 90 sec by using a sonicator (UD-201, TOMY). The debris was removed by filtering the sonicated solution with a glass fiber filter paper and subjected to another round of n-hexane extraction. The collected n-hexane solution was evaporated in a rotary evaporating system (N-1100 and NVC-2100, EYELA). The weight of the residue left in the flask was quantified as total neutral lipid.

### Gene expression profiling

The publicly available RNA-seq data of *E. gracilis*, SRP060591, was retrieved from the NCBI Sequence Read Archive (https://www.ncbi.nlm.nih.gov/sra). The raw reads of the RNA-seq data was trimmed and filtered using Trimmomatic (v.0.36)^38^ with the following settings: LEADING: 20 TRAILING: 20 MINLEN: 50. Then, the clean reads were mapped to the transcriptome assembly data of *E gracilis*, GDJR00000000.1, retrieved from the GenBank, using the BWA program (v. 0.7.17)^39^ with the mem -M setting. The expression abundance of each contig was estimated in the transcriptome assembly based on reads per million (RPM) values. To represent expression pattern of transcripts that encode enzymes involved in the GSH metabolism in *E. gracilis*, contigs homologous to those of *Saccharomyces cerevisiae* were identified by BLAST search with the parameter settings of -v 5 -b 5 -m 9 -F F -e 1e-05, and analyzed expression pattern of their closest transcripts for each enzyme.

## Electronic supplementary material

Supplementary Information

## References

[1] Chisti Y (2007). Biodiesel from microalgae. Biotechnol. Adv..

[2] Brennan L, Owende P (2010). Biofuels from microalgae-A review of technologies for production, processing, and extractions of biofuels and co-products. Renew. Sustain. Energy Rev..

[3] Johnson, L. P. The taxonomy, phylogeny, and evolution of the genus *Euglena*. In *The biology of Euglena, Vol. I*, ed DE Buetow, pp. 1–25. Academic Press Inc., New York (1968).

[4] Zimorski, V., Rauch, C., van Hellemond, J. J., Tielens, A. G. M. & Martin, W. F. The mitochondrion of *Euglena gracilis*. In *Euglena: Biochemistry, Cell and Molecular Biology*, pp. 19–37. Springer (2017). 10.1007/978-3-319-54910-1_2 28429315

[5] Inui H, Miyatake K, Nakano Y, Kitaoka S (1983). Production and Composition of Wax Esters by Fermentation of *Euglena gracilis*. Agric. Biol. Chem..

[6] Suzuki K (2015). Selection and characterization of *Euglena anabaena* var. *minor* as a new candidate Euglena species for industrial application. Biosci. Biotechnol. Biochem..

[7] Suzuki K (2017). Large-Scale Cultivation of *Euglena*. Adv. Exp. Med. Biol..

[8] Yamada K (2016). Efficient selective breeding of live oil-rich *Euglena gracilis* with fluorescence-activated cell sorting. Sci. Rep..

[9] Ogawa T (2015). Enhancement of photosynthetic capacity in *Euglena gracilis* by expression of cyanobacterial fructose-1,6-/sedoheptulose-1,7-bisphosphatase leads to increases in biomass and wax ester production. Biotechnol. Biofuels..

[10] Nakazawa M (2015). Alteration of Wax Ester Content and Composition in *Euglena gracilis* with Gene Silencing of 3-ketoacyl-CoA Thiolase Isozymes. Lipids..

[11] Inui H, Miyatake K, Nakano Y, Kitaoka S (1982). Wax ester fermentation in *Euglena gracilis*. FEBS Lett..

[12] Barsanti L, Passarelli V, Evangelista V, Frassanito AM, Gualtieri P (2011). Chemistry, physico-chemistry and applications linked to biological activities of β-glucans. Nat. Prod. Rep..

[13] Rotte C, Stejskal F, Zhu G, Keithly JS, Martin W (2001). Pyruvate:NADP+oxidoreductase from the mitochondrion of *Euglena gracilis* and from the apicomplexan Cryptosporidium parvum: A biochemical relic linking pyruvate metabolism in mitochondriate and amitochondriate protists. Mol. Biol. Evol..

[14] Inui H, Miyatake K, Nakano Y, Kitaoka S (1984). Fatty acid synthesis in mitochondria of *Euglena gracilis*. Eur. J. Biochem..

[15] Teerawanichpan P, Qiu X (2010). Fatty acyl-coA reductase and wax synthase from *Euglena gracilis* in the biosynthesis of medium-chain wax esters. Lipids..

[16] Tomita Y (2016). Succinate and lactate production from *Euglena gracilis* during dark, anaerobic conditions. Front. Microbiol..

[17] Leustek T, Martin MN, Bick J-A, Davies JP (2000). Pathways and regulation of sulfur metabolism revealed through molecular and genetic studies. Annu. Rev. Plant Physiol. Plant Mol. Biol..

[18] Davies WH, Mercer EI, Goodwin TW (1966). Some observations on the biosynthesis of the plant sulpholipid by *Euglena gracilis*. Biochem. J..

[19] Brunold C, Schiff JA (1976). Studies of sulfate utilization of algae: 15. Enzymes of assimilatory sulfate reduction in *Euglena* and their cellular localization. Plant Physiol..

[20] Schafer FQ, Buettner GR (2001). Redox environment of the cell as viewed through the redox state of the glutathione disulfide/glutathione couple. Free Radic. Biol. Med..

[21] Kawano Y (2015). Involvement of the yciW gene in l-cysteine and l-methionine metabolism in *Escherichia coli*. J. Biosci. Bioeng..

[22] Hao OJ, Chen JM, Huang L, Buglass RL (1996). Sulfate‐reducing bacteria. Crit. Rev. Environ. Sci. Technol..

[23] Hansen TA (1994). Metabolism of sulfate-reducing prokaryotes. Antonie Van Leeuwenhoek..

[24] Kawano, Y., Suzuki, K. & Ohtsu, I. Current understanding of sulfur assimilation metabolism to biosynthesize l-cysteine and recent progress of its fermentative overproduction in microorganisms. *Appl. Microbiol. Biotechnol*. (2018). 10.1007/s00253-018-9246-4 30046857

[25] Ballatori N (2009). Glutathione dysregulation and the etiology and progression of human diseases. Biol. Chem..

[26] Ganguli D, Kumar C, Bachhawat AK (2007). The alternative pathway of glutathione degradation is mediated by a novel protein complex involving three new genes in *Saccharomyces cerevisiae*. Genetics..

[27] Yoshida Y (2016). De novo assembly and comparative transcriptome analysis of *Euglena gracilis* in response to anaerobic conditions. BMC Genomics..

[28] Jiang J (2016). Hydrogen Sulfide-Mechanisms of Toxicity and Development of an Antidote. Sci. Rep..

[29] Mansfield KD, Simon MC, Keith B (2004). Hypoxic reduction in cellular glutathione levels requires mitochondrial reactive oxygen species. J. Appl. Physiol..

[30] Scheer A, Parthier B (1982). Dark-induced chloroplast dedifferentiation in *Euglena gracilis*. Planta..

[31] Christensen JE, Dudley EG, Pederson JA, Steele JL (2016). Peptidases and amino acid catabolism in lactic acid bacteria. Antonie Van Leeuwenhoek..

[32] Barker HA (1981). Amino acid degradation by anaerobic bacteria. Annu. Rev. Biochem..

[33] Carbonero, F., Benefiel. A. C., Alizadeh-Ghamsari, A. H. & Gaskins, H. R. Microbial pathways in colonic sulfur metabolism and links with health and disease. *Front. Physiol*. **3 NOV** (2012). 10.3389/fphys.2012.00448 PMC3508456 23226130

[34] Yin J (2016). l-Cysteine metabolism and its nutritional implications. Mol. Nutr. Food Res..

[35] Cramer, M. & Myers, J. Growth and Photosynthetic Characteristics of *Euglena gracilis*. **17**, 384–402 (1952).

[36] Koren, L. E. & Hutner, S. H. High-yield media for photosynthesizing *Euglena gracilis* Z. *J. Protozool*. **14**, (Suppl.), 17 (1967).

[37] Ayers AL, Dooley JJ (1948). Laboratory extraction of cottonseed with various petroleum hydrocarbons. J. Am. Oil Chem. Soc..

[38] Bolger AM, Lohse M, Usadel B (2014). Trimmomatic: a flexible trimmer for Illumina sequence data. Bioinformatics..

[39] Li H, Durbin R (2009). Fast and accurate short read alignment with Burrows-Wheeler transform. Bioinformatics..

