# Supplementary material for: Characterization of sulfur-compound metabolism underlying wax-ester fermentation in Euglena gracilis
Source: Sci Rep. 2019 Jan 29;9:853. doi: 10.1038/s41598-018-36600-z (PMC6351624; doi:10.1038/s41598-018-36600-z)
Supplement: Supplementary file 1 — Supplementary Information [file 41598_2018_36600_MOESM1_ESM.pdf]

Supplementary Information

Characterization of sulfur-compound metabolism underlying wax-ester fermentation in *Euglena gracilis*

Koji Yamada[1,2], Tomoaki Nitta[1], Kohei Atsuji[1,2], Maeka Shiroyama[3], Komaki Inoue[4], Chieko Higuchi[1], Nobuko Nitta[1], Satoshi Oshiro[3,5], Keiichi Mochida[2,4,6,7], Osamu Iwata[1,2], Iwao Ohtsu[1,3], and Kengo Suzuki[1,2*]

1. *euglena Co., Ltd., Tokyo 108-0014, Japan*
2. *Microalgae Production Control Technology Laboratory, RIKEN, Kanagawa 230-0045, Japan*
3. *Innovation Medical Research Institute, University of Tsukuba, Ibaraki 305-8577, Japan*
4. *Center for Sustainable Resource Science, RIKEN, Kanagawa, 230-0045, Japan*
5. *Department of Bioresources Engineering, National Institute of Technology, Okinawa College, Okinawa 905-2192, Japan*
6. *Kihara Institute for Biological Research, Yokohama City University, Kanagawa, 244-0813, Japan*
7. *Institute of Plant Science and Resources, Okayama University, Okayama, 710-0046, Japan*

*suzuki@euglena.jp

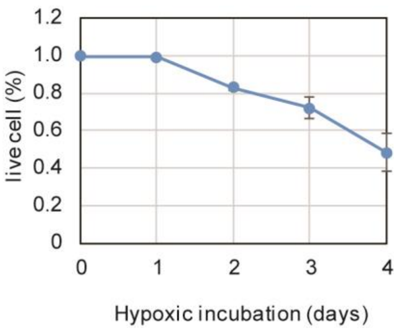

Supplemental Figure 1 Viability of *E. gracilis* cells in hypoxic incubation. The viability of *E. gracilis* cells in hypoxic incubation based on live/dead staining. Hypoxically incubated cells were stained with 0.2 % of trypan blue. Then, the proportion of live cells were determined by counting the unstained live cells and stained dead cells separately. Error bars indicate SD. n=3.

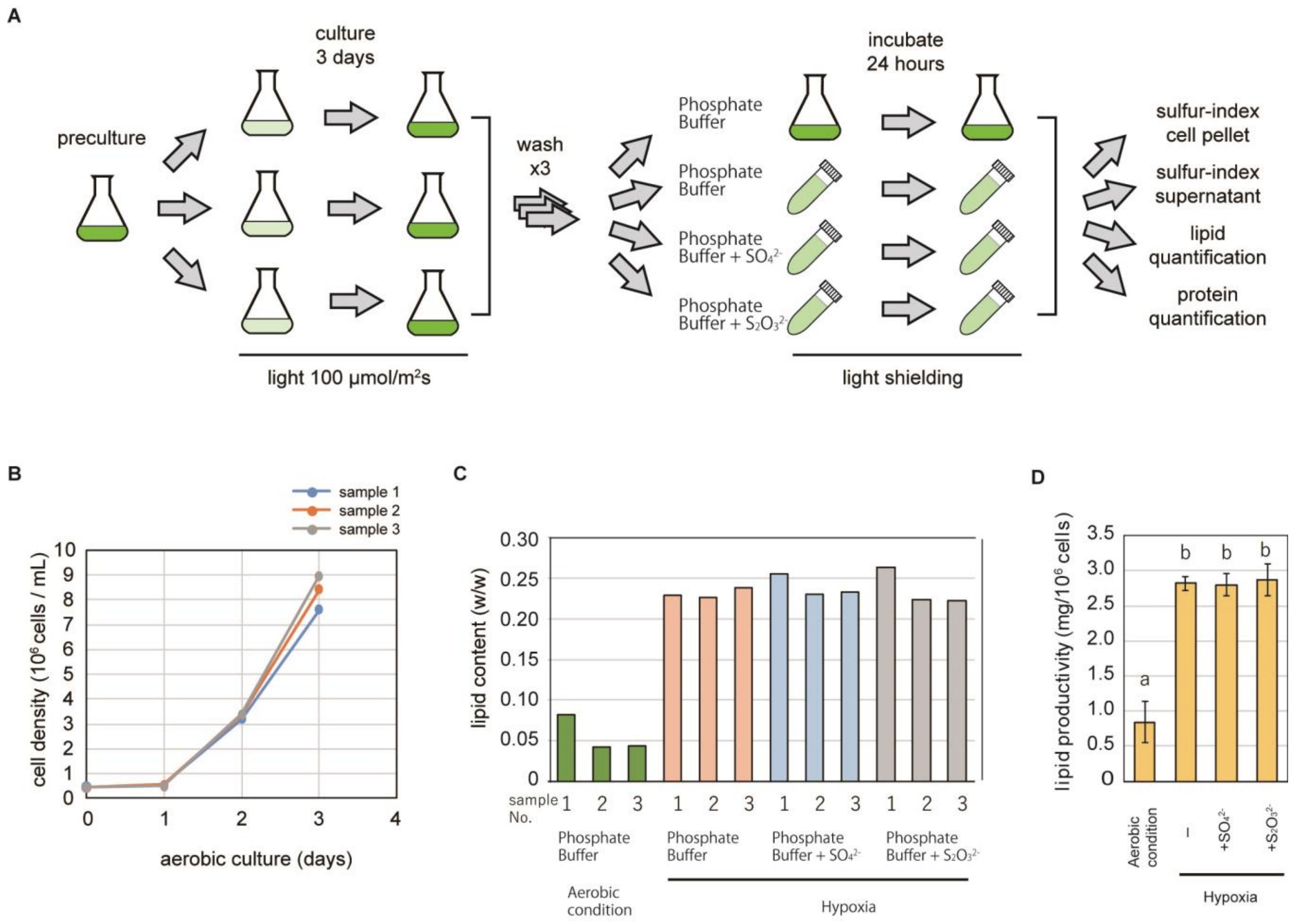

Supplemental Figure 2 Preparation of the samples used in the sulfur-metabolomics. (A) An illustration of culture and subsequent conditioning procedures. Each procedure was performed in triplicate to validate the reproducibility. After 3 days of culture, the cells were washed, divided into 4 samples, and subjected to 24 hours of respective conditioning. Then, the samples were subjected to sulfur-metabolomics on the cell pellet and supernatant, independently, and quantification of lipid and protein. (B) Proliferation curve for each sample in the main cultures. The cell densities of the main culture in triplicate were quantified daily. (C) Lipid content (weight/weight) of each sample. Lipid accumulation in all samples in hypoxia was verified compared to those in aerobic conditions. (D) Mean value of the lipid productivity (mg/10^6 cells) in the experiments. Error bars indicate SD. $n=3$, a, b: means with the different character indicates significant difference, $p < 0.05$ Tukey's multiple test.

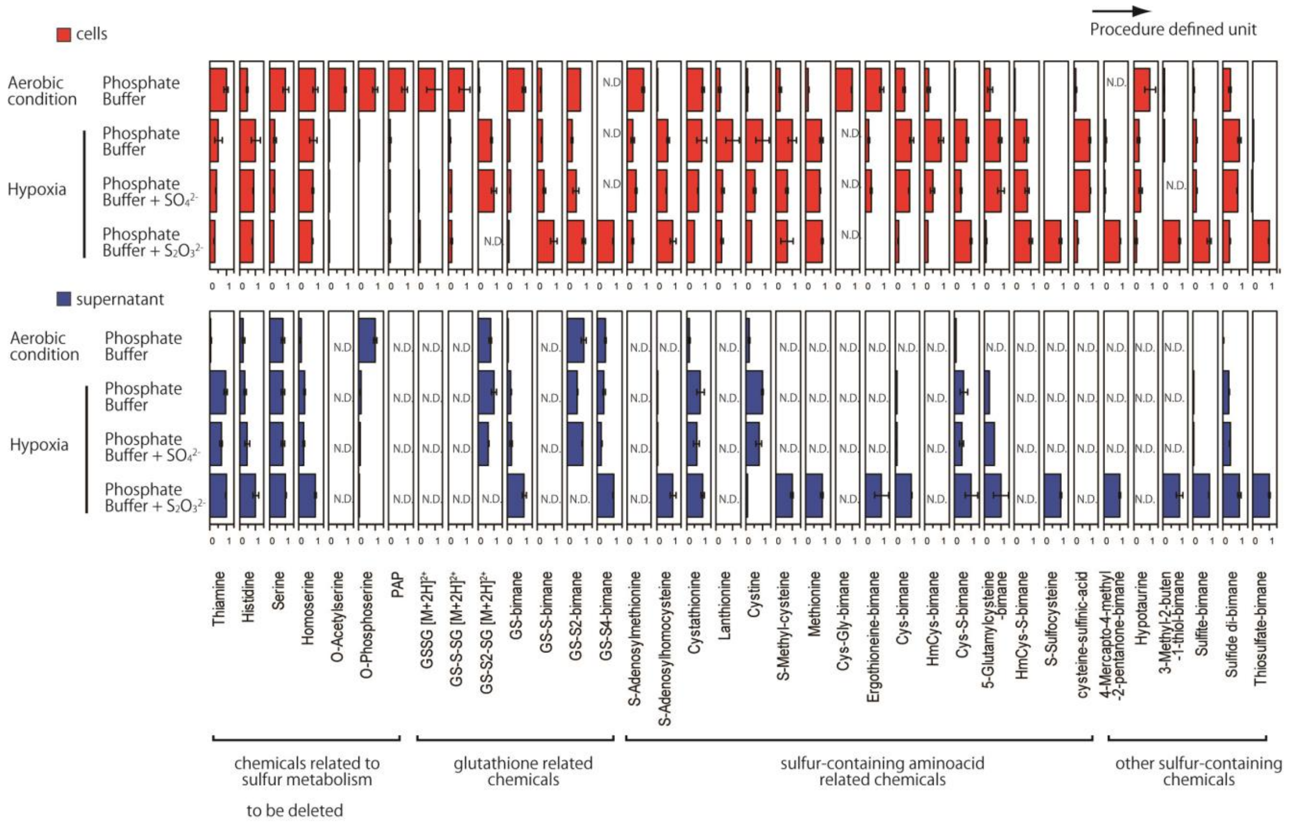

Supplemental Figure 3 Summary of sulfur-metabolomics. Relative quantification of the detected compounds in the LC/MS analysis. The horizontal bars indicate the average of LC/MS signals from analysis in triplicate, which is corrected by the cell number and normalized as the highest amount in the 4 conditions to 1 (procedure defined unit). Error bars indicate SD. N.D. indicates that the compound was not detected in the condition, whereas if the compound was detected in any sample of the triplicate, the average was calculated and displayed in the graph.

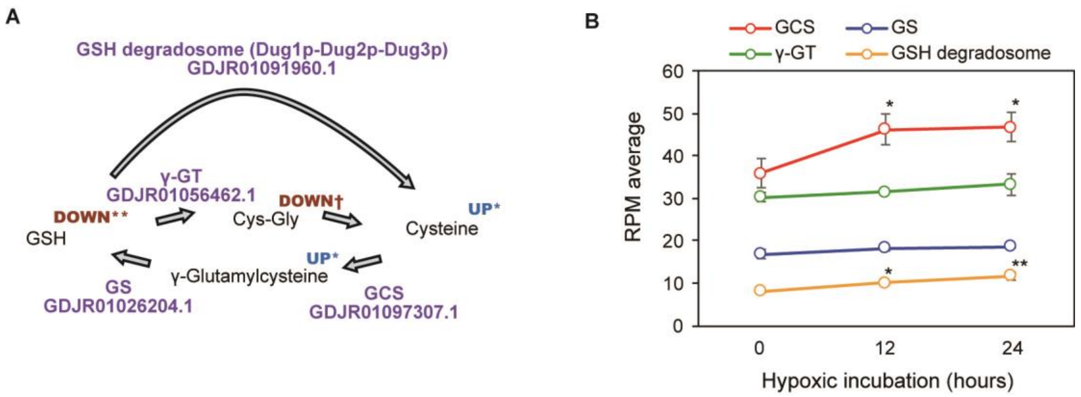

Supplemental Figure 4 Expression of the genes involved in the GSH metabolism. (A) An illustration of the metabolism of GSH. The arrows between compounds indicate metabolic reactions. The name of enzymes which are responsible for the reaction is described on the arrows with that of the identified *E. gracilis* genes. The superscript for each compound indicates the upregulation (UP) or downregulation (DOWN) of the compound with or without a mark indicating the significant difference (*: $p < 0.05$ t-test, **: $p < 0.05$ t-test with Bonferroni's correction, †: compounds are not detected in either of condition). (B) Estimated expression level of the genes involved in the GSH metabolism. The publicly available RNA-seq data of *E. gracilis*, SRP060591, was analyzed to estimate the expression level of the genes in hypoxic (anaerobic) condition based on reads per million (RPM) value. Error bars indicate SD. n=3, *: $p < 0.05$ Dunnett-test, **: $p < 0.05$ Dunnett-test with Bonferroni's correction.
